# Alcohol and social connectedness for new residential university students: implications for alcohol harm reduction

**DOI:** 10.1080/0309877X.2018.1527024

**Published:** 2018-10-18

**Authors:** Rachel Brown, Simon Murphy

**Affiliations:** DECIPHer, Cardiff University, Cardiff, United Kingdom

**Keywords:** alcohol, university, social connectedness, qualitative, education

## Abstract

Starting university is a significant life-event, commonly involving detachment from existing social networks and emotional stresses that increase risk of drop-out. The developmental need to form new peer relationships is prominent during this period and is correlated with successful adaptation. This study investigated the role of alcohol in the process of transition and peer group development for new students. Thematic analysis of semi-structured interviews is presented, conducted within a broader instrumental case study of campus approaches to alcohol policy and management. Twenty-three first-year students participated in interviews lasting between 45–60 minutes. Verbatim transcription was followed by within- and cross-case analysis. Drawing on social connectedness theory, we illustrate how pre-arrival concern over new peer relationships was subsequently reduced by drinking together. This reinforced participant perceptions of alcohol as beneficial for hastening development of social connections, in turn reducing anxiety and supporting successful transition. For non-/low-drinkers in the study, social connectedness without alcohol use was reported as more challenging. Alcohol was perceived as a readily-available, effective tool for hastening social connectedness, increasing student resistance to alcohol education messages provided at the start of term. Implications for addressing alcohol-related harms in students are discussed.

## Introduction

The transition from home to university represents a space requiring active navigation by new students (Palmer, O’Kane, and Owens 2009). Although transition presents opportunities for personal growth, it is also associated with psychological change and increased stress (Dyson and Renk 2006; Fisher and Hood 1987) arising from separation from support networks (Rice 1992). Loss of links to existing peer groups (Ellison, Steinfield, and Lampe 2007) can challenge adaptation to university life (Paul and Brier 2001), with the new emotional demands placed on the transitioning student threatening adjustment and increasing risk of leaving prematurely (McMillan 2013).

Tinto’s (1975) model of student retention states that successful transition requires both social and academic integration, with social integration correlated with the acquisition of a friendship group, positive relations with staff and reported level of enjoyment at university. Integration into a larger social context further requires perceived social connectedness, associated with the feeling of belonging and relatedness with people and stemming from the quantity and quality of connections between self and others (Lee and Robbins 1995). Social connectedness is associated with lower perceived stress among students (Lee, Keough, and Sexton 2002) and is considered a precursor to perceived social support (Mashek et al. 2006). This has in turn been identified as a significant factor in reducing risk of student drop-out (Mallinckrodt 1998; Nicpon et al. 2006).

Alcohol is seen as an important social lubricant in UK student populations (Read et al. 2003; Seaman and Ikegwuonu 2011), and may be influential during the process of transition to university through its role in facilitation of social group formation. For a majority of UK students, university co-occurs with the attainment of legal drinker status and increased exposure to alcohol consumption, both among peers and in drinking environments (Biederman et al. 2000; Rosenquist et al. 2010). This may increase exposure to risk of harm, with heavy drinking associated with multiple adverse outcomes in student populations, including accidents (Clapp, Shillington, and Segars 2000), being a victim of crime (Newbury-Birch et al. 2009) and increased risk of unprotected sex (White and Hingson 2013).

Evidence suggests that large numbers of students are consuming at levels considerably over recommended guideline amounts and frequently in binge-style drinking episodes (Craigs et al. 2011; Morton and Tighe 2011). Expectations of drinking as a normalised part of student behaviour are widely-held (Hebden et al. 2015), with deviations from stereotypical norms viewed as strange or unsociable (Conroy and de Visser 2012). The associated risks of student drinking therefore suggest an ongoing need to challenge alcohol norms and behaviours on campus.

Although alcohol awareness and safe drinking campaigns are commonly undertaken at UK universities (Orme and Coghill 2014), such approaches show little impact on consumption (Larimer and Cronce 2007). Students are likely to disregard advice from universities in favour of making their own decisions as part of newly-acquired adult status (Snow et al. 2003). Further, alcohol awareness efforts delivered in drinking settings are often considered incongruent with the perceived purpose of the setting (Brooks 2011).

Resistance to change may also be more likely where there are perceived social benefits of alcohol, particularly during the, potentially challenging, transition period. Drinking together is perceived as important in the process of social bonding at university (Kairouz et al. 2002; Read et al. 2003), with peer drinking levels rated as the most significant reason for own drinking levels by students (Faulkner et al. 2006). Integration into heavy-drinking peer groups is strongly associated with increased consumption (Cheng and Lo 2015), and deviation from peer group norms is increasingly difficult with greater degree of integration (Hornsey et al. 2007). This suggests that, should early peer group development in students be formulated around alcohol, heavier drinking norms will be more difficult to reduce later where group behaviour has been established. Evidence suggests that the desire to drink in moderation is a strong predictor of future behaviour (Fry et al. 2014), but this desire may be less likely to develop if benefits from non-moderate drinking are obtained, including social connections.

For universities to more effectively intervene in reducing harms associated with student alcohol use, better understanding of the range of drivers to current behaviour is important. This includes the perceived benefits attained from drinking among student cohorts. If alcohol acts to facilitate the development of new peer relationships and, consequently, reduces transition anxiety through meeting needs for social connections, this has implications for addressing harms associated with consumption in this population. Further, this may suggest that non-/low-drinking students are at risk of social exclusion as a result of non-participation in traditional drinking activities.

This article explores alcohol use as an aid to social integration for a sample of new, residential students at one UK university. It reports findings from qualitative research exploring the role of alcohol in relationship development and discusses the risks of harm associated with this potential perceived benefit. It also discusses the role of the university setting in facilitating continuation of heavy drinking norms.

## Methods

This research explored student perceptions of alcohol as a means of reducing transition anxiety and aiding social connectedness. Data is reported from in-depth, semi-structured interviews with first-year students (*N* = 23) at one urban, UK university. Ethical approval for the research was gained from the institution’s research ethics board. Interviews were carried out as part of a broader, instrumental case study (Stake 1995) exploring the development of alcohol policy and practice at the site. Within an instrumental case study, case selection is not based on either unique features or representativeness, but on opportunity to study the research problem by identifying features that may be relevant in other settings (Bassey 1999). The case should not be considered a ‘typical’ example of a university, with concepts of typicality deemed problematic in social settings where each possesses unique processes and interactions (Stake 2000). Case study here provides opportunities to elicit context-dependent knowledge, which foregrounds the development of broader principles (Flyvbjerg 2006).

All interviews were conducted by the lead author, with discussion of pre-arrival concerns, expectations of alcohol use and post-arrival drinking behaviour. We further explored the perceived role of alcohol in relationship formation and the significance of this for adaptation. Interviews took around 45–60 minutes and were held in a private room in the Student Union (SU), as chosen by all participants from several available options. The semi-structured approach facilitated personal, non-prescriptive accounts of significant issues, allowing flexibility in exploring new and emerging avenues (Newton 2010) and the inclusion of theoretically-derived themes (King and Horrocks 2010).

### Mapping the research setting

Development of student interview guide content was facilitated through in-depth exploration of organisational processes impacting student experiences, including analysis of documents relating to alcohol, and observations of campus harm-reduction activities. Two site visits were made (with permission) to key areas including bars and housing, and any visible material relating to alcohol was noted. This included awareness-raising posters, rules/policies and signposting to advice services. All policies mentioning alcohol were also obtained on request and were analysed to consider: embedded rules, target audience, structure and organisation and inclusion of evidence (Rapley 2008). University webpages were searched for references to alcohol and the context of this noted, including any advice/guidance and any reported prohibitions. Interviews were also carried out with staff (*N* = 17) to understand the development of alcohol policy and practice, with findings from these also contributing to the student interview guide. These staff were identified through discussion with human resources, as well as the lead researcher’s own previous experience of delivering alcohol work in university settings, meaning familiarity with key departments. All were from non-academic teams with direct involvement in student well-being, including staff from Student Residences, Student Support and the Student Union. Within these teams interviews were carried out with those self-defining as involved in alcohol policies and/or practice development at the time of interview. From this mapping activity, questions were formulated for interviews with students, to include awareness and perceptions of the range and content of activities noted as well as perceptions of and behaviours around alcohol.

### Participants

Students cannot be considered as officially attending any specific university prior to registration, meaning no pre-/post-test interview design was possible to explore views and behaviour prior to joining. Instead, first-year students were targeted in the first term to ensure that reflections on transition were as recent as possible. First-year residential students were selected due to evidence of heavier drinking among those living on-campus away from family supervision (Thombs et al. 2009; Ward and Gryczynski 2009). Concern over formation of new peer relationships is also higher among this group compared with students commuting from the family home (Buote et al. 2007). University data protection policy meant that it was not possible to contact students directly through emails or residential addresses; therefore, on the recommendation of staff participants, recruitment was carried out through face-to-face contact outside of residences sites, where the research was outlined, including the offer of a £10 shopping voucher for participants. This was supplemented through a convenience sampling method, utilising the lead author’s part-time teaching work. This direct approach is commonly utilised in organisational studies where the researcher may be in a position to access a population due to their own role (Bryman 2008). It was important that students felt no pressure or coercion due to the power differential inherent in the institutional relationship (Miller and Bell 2002), therefore they were asked only if they would be willing to take flyers back to their accommodation and display them somewhere visible to flatmates. Subsequently, some who agreed to this did ask to take part themselves and were accepted into the study. Twenty-one interviewees lived in university-owned halls and two lived in the same shared house off-campus. Participants may be ‘typical’ according to the age and residential profile of a majority of UK undergraduate students but are not here being defined as representative, reflecting the qualitative nature of the study (Barbour 2001). Findings are applicable to this group but may provide insights into experiences of others within similar settings.

### Data analysis

Thematic analysis (Braun and Clarke 2006) was used to explore the data, commencing with initial reading by the primary author for emerging themes. An iterative coding framework was developed from this reading, then enhanced through re-reading and through consideration of relevant research literature. This allowed for incorporation of emerging and unplanned interview data that is, arguably, unsuitable for inter-rater coding (Morse 1997). Inter-rater reliability was not available for this thesis project; however, code development was discussed with the study co-author at multiple points. All decisions on collapsing and refining codes were rationalised and agreed with the study co-author prior to final development of content categories. These were centred on student anxieties prior to arrival at university; expectations of alcohol use; and interpretations of the role and function of alcohol in relationship processes. The flexibility of the thematic analytic approach is evident where the focus is on depth of meaning within the data (King and Horrocks 2010). Illustrative quotes are presented where they are indicative of emergent themes from the data. Throughout the article students are referred to as S(n). Summary biographical information is provided in Table 1.

**Table 1.** Student participants.

	Personal details
S1	White English female, 18 years old, studying History
S2	White English male, 19 years old, studying Criminology
S3	White English male, 18 years old, studying English
S4	White Welsh female, 19 years old, studying Chemistry
S5	White English female, 18 years old, studying Optometry
S6	Female EU student (did not want country of origin recorded), 19 years old, studying Maths
S7	Asian female International student, 19 years old, studying Psychology
S8	White Welsh female, 20 years old, studying Criminology
S9	White Welsh male, 20 years old, studying Biomedical Science
S10	White English male, 19 years old, studying Geosciences
S11	White English female, 18 years old, studying Music
S12	White male EU student, 20 years old, studying Engineering
S13	White English female, 19 years old, studying Sociology
S14	White English female, 21 years old, studying Criminology
S15	Asian Welsh female, 19 years old, studying Education
S16	White English male, 20 years old, studying Pharmacy
S17	White English male, 19 years old, studying Chemistry
S18	White English female, 19 years old, studying Criminology
S19	White Welsh female, 18 years old, studying Psychology
S20	White Welsh female, 19 years old, studying Education
S21	Black English male, 19 years old, studying Pharmacy
S22	White English male, 18 years old, studying Computer Science
S23	White English male, 19 years old, studying Biomedical Science

## Results

### Main worries before arrival

We asked students to reflect back on what they had been most worried about in the summer directly before starting university. For most, the pressure to develop new friendships was their main source of anxiety, with concern focused primarily on new living companions rather than on academic coursemates. Although this worry was consistent across the group, with no patterning by gender, age or course of study, these kinds of concern were not the main focus of transition activities on-site. Mapping and staff interviews identified that transition support at the university was primarily focused on academic and life skills development, such as financial planning, and largely aimed at sub-groups of the student population, such as those without family support, ex-service personnel and others. The university did feature a mentoring service for the general new student population, which included advice from older students on societies available, local facilities and academic support. This therefore offered some social support potential, but was available to a very small fraction of the first-year population on current resourcing. This means that, for a majority, the structure of arrival sees first encounters with new housemates happen before contact with other aspects of the university, such as staff structure, academic structure or coursemates. These encounters were therefore seen as highly significant for initial integration:

You don’t want to be the one who’s like not even happy in your flat or you don’t bond with anyone properly in your flat because you’re stuck with them … if you were just unhappy, I don’t think you’d last very long. (S4, F)

If you’ll meet people who you’ll get along with or if you’ll like struggle to make friends and stuff … because everyone says it’s easy to make friends but you don’t actually know what it’s going to be like until you get there. (S9, M)

Social connectedness is associated with positive appraisal of environment (Fox and Marsh 1998), and this was seen here in participant expectations that absence of bonds would potentially impact their attitudes towards the wider university experience:

I think obviously you’re worried about like getting on with people like making friends and stuff; you know that’s the last thing you want is to come here and not. (S3, M)

I think I just knew if I didn’t get along with people I just would hate everything else about uni. (S1, F)

Some students reported taking steps to reduce these pre-arrival concerns, going online to either Facebook groups linking students from the same residences or the chat site ‘Student Room’, where current and previous students post information on halls, social activities in the area and other aspects of university life. This generally involved planning drinking events, with previous residents suggesting ‘big pre-drinks’ sessions to meet housemates and break the ice on arrival. Online groups like this were identified as helpful in starting to create an image of campus life and also providing opportunities for development of social connections; for example:

Definitely Facebook was a big part … we all talked over Facebook, tried to get to know each other a little bit more so that was nice, that was really good. (S11, F)

For participants, these sites were generally viewed as a positive; however, the emphasis on planning social events around alcohol may act to develop and reinforce the positioning of alcohol as normative at university, with little evident challenge to this from other users of the site.

### Alcohol and social connections

Pre-university drinking experience was highly varied among participants, with a majority having drunk at least occasionally before but most citing that their consumption had increased significantly on arrival. Prior experience of alcohol as a social lubricant within peer relationships likely contributed to reported expectations of alcohol as helpful in bonding at university. For several participants, this was further reinforced through the experiences of parents, older siblings or peers who had gone to university previously and shared their drinking experiences, strengthening their association between student status and alcohol. For example:

You’ve got like cousins and friends and stuff who have gone to uni and, sort of like, they’ve been saying you drink quite a lot at uni and stuff to relax and socialise. (S22, M)

My brother’s three years older than me and he’s always talking about that [drinking at university]. (S13, F)

Pre-arrival online activity, previous personal experience and interpersonal relationships acted to develop and reinforce expectations that post-arrival social integration would involve drinking. This was then widely reinforced by initial experience of the alcohol-intense university and local community context on arrival. At this site, and others in the UK, the Freshers period characterising the beginnings of university life has historically embedded associations with alcohol use (Fuller et al. 2017). It was notable that these were not officially expressed, with SU staff keen to communicate that none of their publicity materials for Freshers mentioned alcohol. This contrasted heavily with flyers and promotional material available everywhere around campus from local bars and clubs, which focused predominantly on drink promotions. However, student and staff expectations, based on social norms, inter-personal messages and experiences, illustrate that, regardless of whether alcohol is stated explicitly as part of social activity, this is assumed anyway. It was commonly expected here that drinking would be the primary social activity for new arrivals:

You know that basically Freshers is just going to be, just total binge drinking chaos really. (S4, F)

It’s synonymous with Freshers week. I went round to see all my friends at different unis so like, I’d spent weekends in the different ones and it’s quite a common theme, like it’s what everyone does to tolerate each other. (S16, M)

This was echoed in staff perceptions:

At the end of the day there’s like two and a half thousand 18 year olds really so there’s going to be a lot of them that are going to want to go out and drink and there’s nothing you can do about that. (SU staff, M)

This normative understanding of the likely dominance of alcohol during initial university life was shared regardless of own drinking experience and behaviour, with self-defined non-/low-drinkers as likely to cite this view, illustrating the dominance of stereotypical portrayals. The few non-/low-drinkers included here reported planning numerous tactics for this period in the knowledge that they were likely to be excluded from typical activities, including securing part-time work and arranging multiple visits from family to keep busy. These adaptations illustrate the potential for the alcohol-intense Freshers period to socially exclude some new students, with implications for their experience of transition.

To understand how this period had been negotiated by self-reported drinking students, we discussed behaviour after arrival, including tactics employed for social engagement and the role of alcohol within these processes. For most, the prior expectation of drinking together was enacted through consumption in flats on initial meetings as a means of developing connections. Several described bringing special bottles of spirits to share, planning alcohol shopping trips together and planning drinking games as means of overcoming initial tensions:

It was the first night we had in our halls; they’d put a board game on the table and it was, if you land on this you have to do certain things and one of the guys like had to eat a raw onion and just random things like that and then it got a bit rubbish so we played Ring of Fire [*drinking game*]. (S5, F)

High-risk drinking, including drinking games, was widely reported as being common during initial social activity with new peers. Drinking games are perceived within groups as socially beneficial (Workman 2001), potentially due to the associated reduction of inhibitions but also as an expression of trust in new contacts. However, they are also associated with increased consumption and risk of negative outcomes (Clapp, Reed, and Ruderman 2014). No concern over this was reported, with drinking together observed as positively impacting and enhancing commonality, as a precursor to the development of social connectedness:

Alcohol probably does make it easier, takes away the awkwardness of it, I think. You can have something in common when you’re drinking with people. (S2, M)

I think in the first week, settling in, having to live with people that you don’t know, it’s a lot easier to get to know them by having a bit [*of alcohol*]. (S14, F)

It sounds stupid but you kind of feel a bit more confident when you’ve had a drink or something. So at the start of pre-drinks on the first night I was just standing in the corner like ‘what am I doing here? I don’t know anyone.’ (S8, F)

Some students drew a distinction between drinking together and chatting in halls as ‘doing something’, as opposed to chatting without alcohol as ‘doing nothing’, reflecting anthropological accounts of alcohol as a marker used to characterise a situation as a social event (Fox and Marsh 1998):

I think that the drinking helps with like meeting your neighbours as well … because you’re more likely to be in and out … whereas to go and knock on their door in the afternoon for a cup of tea … it doesn’t seem the same as for drinking. (S4, F)

It also gives you an excuse just to sit around and talk, which like, just sitting in the kitchen not having a drink, chatting just seems a bit strange with nothing to put your hands on. (S1, F)

Students are highly likely to consider alcohol as a social tool aiding friendship formation (Collins et al. 2014) and this was reflected in the data, with most who drank rating this as aiding friendship development, through both confirmation of shared norms and in reducing anxiety:

Because like loads of your peers do it and if you want to make friends you do it as well, yeah. (S7, F)

The first week we pretty much went out every single night … I think that did help like you find out things about each other. (S1, F)

A lot of people are quite nervous about meeting new people and they just find a little bit of drink and seeing other people drinking and you just get a bit more confident… (S9, M)

The development of shared bonds, including stories and experiences, indicates the beginning of a shift from being individuals in a shared space to the attainment of social group status (Deaux 2001). This process was observed here, with alcohol rated as highly significant to group development by a majority, reflecting internalisation of cultural understandings associating sociability with alcohol use (Griffin et al. 2009):

Probably the relationship wouldn’t have been as strong. There is something about going out with people when they’re getting drunk and having a good time that does, sort of, bring you close together… (S23, M)

Positive appraisal of peer groups is significant in perceived social connectedness (Lee, Draper, and Lee 2001). This was observed here through the reported effect of alcohol in reducing inhibitions, which aided initial bonding and also enhanced feelings of safety provided by group membership (Baron and Kerr 1992):

I think, yeah, it just relaxes you a bit and then you start having a bit more fun and I guess, see who the silly ones are and things like that when you get a bit more drunk… (S4, F)

But then after you’ve had a few nights and stuff you get to know people and it’s like when something happens, like if something bad happens on a night out and someone helps you, you know like who you can trust and stuff as well. (S8, F)

Although students generally stated that bonding with new peer groups would have happened eventually, it was felt by a majority that it happened faster where alcohol was utilised:

I think in the morning when we woke up and we’d all been out together we were like sober again but the awkwardness was like gone, so we got on pretty much straight away from that I think. (S1, F)

In the first night you go out you kind of get to know people properly; obviously you’re, kind of, a bit more relaxed when you do have alcohol … It’s just weird to think what it would have been like without alcohol. (S2, M)

The role of alcohol in enhancing social connectedness is especially significant where recent loss of existing social relationships has occurred and the need to feel embedded in new social contexts is paramount:

I think it [alcohol] probably makes bonding quicker because then you have stories to tell rather than, like … you make your stories with them rather than just telling them about friends from home. (S13, F)

In the context of transition to university, where achievement of social integration through the acquisition of new social connections can impact on the overall success of transition, this importance is magnified further. Here, participants were highly likely to rate alcohol as beneficial to meeting their social needs, and it is notable that no one cited concerns over alcohol-related harms or negative drinking outcomes. This suggests that perceived social benefits were being prioritised over risks among this cohort at this point in time.

### Deviation from the norm

A significant aspect of social connectedness involves feeling accepted as an independent self in relation to others (Lee, Draper, and Lee 2001) and this was impacted here for some students by deviation from majority drinking norms. Three students who defined themselves as non-/low-drinkers all experienced barriers to social connectedness as a result of this, including the feeling of having to explain non-participation as well as perceiving that formation of peer groups had taken longer for them than for drinking peers. There were observable differences in the framing of alcohol within friendship processes between students who identified as low- or non-consumers prior to arrival, and students who expected to drink. Students without strong drinker identities were likely to report alcohol as unimportant in their peer group formation but were more likely to report initial difficulties in establishing friendships as well as perceived pressure:

Some people will say like ‘oh well yeah you’d have more fun if you drank’ … there is pressure, people, some people aren’t understanding at all. (S15, F)

The impact of non-adherence to majority norms on alcohol supports the argument that adopting ‘typical’ drinker behaviour constitutes the easy option (Hepworth et al. 2016) for new students in meeting the need for connectedness. All three reported that the process of establishing social connections felt slower for them than for their drinking peers, reinforcing the difficulties associated with deviation from localised standards of behaviour. For S6, this even resulted in having to change accommodation to escape from perceived bullying over non-participation in drinking norms within her flat. Although the SU offers events advertised as ‘alcohol-free’, these occur during the day with nothing in the evening, meaning programming creates temporal segregation between drinking and non-drinking or moderate-drinking students. This segments the use of the SU as a space based around the nature of social activity occurring, thus creating rules of attendance which are then communicated to new students. The SU recognises its role in providing social opportunities to new students:

I think that there is enormous benefit in us running the Freshers offering that we do, way over and above just the income generation that it gives. What we offer is a chance to make friends, and that is what students crave. (SU staff, M)

This suggests that current provision – which is perceived as alcohol-driven – may deter non-drinkers from full participation in the social activities on offer, potentially inhibiting opportunity for social group formation. This was acknowledged by other staff:

It’s a kind of stereotyped cliché that students come, they get pissed all week. And some people come and they don’t actually want to do that. And it is very much geared in the union to go drinking, so I can imagine it is difficult. (Student Support staff, F)

### Responses to campus harm reduction

At this site it was university practice to signpost all new students to webpages on alcohol safety as part of induction and the content of these was discussed in interview. Content, which was written by university staff in Residences, included tips for a safe night out, warnings over problems stemming from excess alcohol use and strategies to reduce consumption. Most students did not recall seeing the webpages so were shown content and asked for their thoughts. The perceived paternalism of warnings and consumption-reduction messages was widely rejected and seen as unrealistic for students, reflecting findings that this population generally sees little need to reduce drinking (Roche and Watt 1999):

This is like the same sort of thing that’s drummed into you at school, like drugs are bad, don’t drink too much, things like that … But things like, I don’t know, the psychological consequences, people aren’t going to care about them and social consequences. (S17, M)

You get taught like in school, in PHSE, not to drink too much but … don’t know, I don’t think it would really alter many people’s behaviour unless they had a real problem where they like depended on it. (S13, F)

For one interviewee, prioritisation of alcohol and sociability negated the value of tips on reducing risky drinking styles:

I think that’s the best way of doing it, personally … I understand that point, though, of you may feel pressured into drinking but I think it’s quite a good social thing to do, rounds. (S12, M)

Where the university stated more specific safety tips around safe travel and guarding possessions, these were more welcome and considered more useful by a majority; however, there was little expectation that such messages would be effective. This expectation was shared by several staff members who commented that, although it was seen as part of the duty of the university to provide such information, it was important to not become paternalistic:

This is not a school, this is a university, you’re dealing with adults. As long as we are, I think, giving students the right advice, the right support, then, then that probably is sufficient. (University Management Team, M)

Where advice contrasts with student conceptions of their own adult status, this is likely to be rejected as contradicting their right to learn from their own experience (Brown and Murphy 2018). Further, such messaging would struggle to compete with established social narratives:

It’s just trying to find the best way of getting messages across really, because we’re not going to change it completely, it’s part of growing up, it’s part of being a young person, let alone a student. (Estates Staff, M)

## Discussion

Alcohol consumption has been described anthropologically as an inherently social act (Jayne, Valentine, and Holloway 2011), with shared consumption assigned high value within the context of peer groups as a tool for cementing bonds (Miles, Cliff, and Burr 1998; de Visser et al. 2013). This research adds to current understanding of the perceived benefits of drinking together in attainment of social integration for new university students. Findings suggest that social motivations to drink are reinforced in this population by the associated reduction in inter-personal anxiety achieved through meeting developmental needs for peer group acquisition, with implications for moderating practices where benefits may be perceived as outweighing costs.

Reflecting previous transition research, the primary anxiety among students before arrival concerned the development of new friendships due to loss of proximity to existing support (Ellison, Steinfield, and Lampe 2007; Rice 1992). This, coupled with pre-arrival expectations of the positioning of alcohol in student life, ensured that bonding through drinking was commonly pursued and widely deemed to be effective. Despite no active alcohol promotion by the SU being the ‘formal’ on-campus message, alcohol is still heavily promoted by local retailers and dominates the informal narrative of Freshers, making the decision to omit it almost redundant. New students reported utilising alcohol as a means of developing social connections with new peer networks and forming bonds. Drinking with housemates was commonly cited as enhancing speed of bonding in a situation where rapid adaptation and acquisition of peer networks is prioritised (Buote et al. 2007; Tinto 1975). The role of alcohol in expediting connectedness served an important function in attainment of social integration, with little to suggest that participants were concerned about alcohol-related risk of harm where social benefits were prioritised.

The dominance of alcohol in stereotypical portrayals of student life was reflected in pre-arrival expectations that alcohol would be influential in relationship development. Although previous drinking experiences were highly variable, the expectation of the centrality of alcohol to university social activity was universal. Previous positive experiences alone cannot account for this shared perspective and instead the dominance of wider societal portrayals of youth – and specifically student – drinking are influential. These may be reinforced by social media activity, by traditional conceptions of Freshers evident in staff and student expectations, which emphasise the centrality of alcohol. After arrival, and despite official narratives, participant alcohol expectations were then met by early experiences, including the nature of Freshers provision, which was based on a period of segmented social events incorporating heavy drinking as the primary evening social activity. Other initial opportunities for social integration were largely student-led and informal; for example, in social events arranged in residences, with little input from the university, leaving space for the commonality of drinking expectations to be met. Although the site offered transition support through mentoring, as well as daytime non-alcohol activities in the SU, these were not mentioned by participants. There was little sense of an alternative option, with those deviating from the norm more likely to report avoidance of social activities, reinforcing that conformity with majority alcohol behaviour may constitute the easier option for meeting social needs. Students are presented with drinking ‘rules’ and norms for the setting through contact with institutional practices (Chatterton 1999). Learning these rules not only reinforces pre-arrival expectations of alcohol and its positioning at university, but also enhances feelings of gaining control of the new situation (Raffo and Reeves 2000), which is significant in minimising the stresses of displacement from existing networks.

However, this absence of challenge to situated norms means that maintenance of existing drinking is likely and exposure to potential alcohol-related harms will continue. This includes exposure to higher risk home drinking as well as games, as reported by this cohort. Pre-drinks in halls of residence increase the likelihood of participation in drinking games (Zamboanga et al. 2014), where high-risk consumption practices are often initiated by the heaviest drinkers in the setting and result in others feeling pressured to take part (Polizzotto et al. 2007). Further, first-year students report higher consumption during drinking games than traditional non-home drinking events, as well as increased experience of adverse consequences (Clapp, Reed, and Ruderman 2014), including blackouts (Ray et al. 2014). Those who more frequently observe drinking games occurring are more likely to participate in them (Johnson, Wendel, and Hamilton 1998). As evidence indicates that drinkers learn to associate particular venues with drinking styles (Seaman and Ikegwuonu 2010), this process of early exposure to alcohol in residences has implications for attempts to challenge alcohol behaviour in student residential settings, with normative perceptions of alcohol use difficult to challenge once established within groups (Livingstone et al. 2011). Further, where a setting is perceived as permissive of risky drinking styles, these styles are more likely to be expressed (Ahern et al. 2008).

Current approaches to alcohol awareness on UK campuses frequently focus on promotion of ‘sensible’ drinking, underpinned by individualised conceptions of drinking as lifestyle choice (Larimer and Cronce 2007). There is little evidence of the impact on high-risk drinking of these approaches (Croom et al. 2010; Paschall et al. 2011), which are potentially incongruent with the strongly social motives underpinning much student consumption. The benefits of drinking together, coupled with reported resistance to alcohol education messages reminiscent of secondary education, mean that the use of safe drinking messages commonly seen in UK campuses is likely to be ineffective (Brown and Murphy 2018). The benefits of shared alcohol norms – for example, allowing first years to begin feeling like students (Banister and Piacentini 2006) – and ease of conformity to dominant cultural narratives and ready provision of alcohol, suggest that drinking is likely to continue where it is beneficial to adaptation to the university experience. Current practice reinforces a cyclical relationship between student identity ↔ provision of drinking spaces ↔ student drinking styles, which is re-enacted on campuses throughout the UK, providing considerable economic benefits to Student Unions and local communities. Any regulation of student alcohol use is therefore being attempted within a setting where it contrasts with both informal narratives and economic benefits to business, with campus environments currently limited in the extent to which they are health promoting (Orme and Coghill 2014).

Messaging around the social risk of excess drinking (de Visser et al. 2013), as well as provision of other opportunities for achieving social integration, including more structured activities at the start of term, may be more acceptable to students and have the secondary effect of reducing emphasis on alcohol. This may be of further benefit to low- or non-drinking young people, for whom the current social offering based heavily on alcohol is unlikely to meet their needs as effectively. For those not conforming to majority alcohol norms, connectedness – and subsequent social integration – is more challenging. Although non-/low-drinking young people may see their own behaviour as a positive, personal life choice (Herring, Bayley, and Hurcombe 2014), this does not preclude social losses, particularly in a context where drinking together is normalised. In light of the recent trend of reduced youth consumption and growing numbers of non-/low-drinkers (Bhattacharya 2016; Institute of Alcohol Studies 2013), it is arguable that universities have an opportunity, and a responsibility, to consider social provision outside of accepted models.

In considering the role of universities in moderating student drinking, this research contributes to the necessary debate on the potential for intervention to reduce harms associated with high levels of consumption in student populations. Current moderation efforts are likely to be significantly limited where student developmental needs are being met through current, high-consumption, behaviour despite its potential for longer-term health risks. The problematisation of student drinking evident in policy approaches (Measham and Brain 2005) contrasts with student experiences of the social benefits underpinning consumption in peer groups and the associated positive outcomes of this during transition. It further contrasts with the normalisation of alcohol in the university setting, with usual practice enabling alcohol use (Thombs et al. 2009) and ensuring that structure supports the agentic response (drinking) of students to inter-personal challenges. Further, current approaches of no direct mention of drinking in SU promotional material are somewhat passive and do not constitute an active challenge to situated norms, meaning they are likely to have limited effect.

As integration is highly significant in this life-stage, the benefits obtained from alcohol use would suggest that high levels of consumption are difficult to change significantly unless alternative means of social integration are available. University approaches to transition, including the construction of post-arrival processes governing the presentation of bonding opportunities, should be further examined, including challenges to the situated norms of student social behaviour and presentations of alternative means to attain goals of adaptation. Although, understandably, focusing transition activities on academic issues, this is not actually reflective of the primary concerns of new arrivals. The generation of new insights about the role of alcohol as a mechanism to meet developmental needs during this process of transition to university can inform both transition strategies and organisational approaches to moderation of negative alcohol outcomes.

### Limitations and future research

Although the case study approach facilitated understanding of the intersection of organisational processes with student behaviour, this also limits generalisability of findings. Further research across a range of campus sizes and locations is recommended. The scale of the study also prohibited in-depth consideration of the process of peer group formation in non-drinking students. Exploring barriers to and facilitators of peer group formation for non-drinkers would further clarify the function of alcohol during this period and the potential impact on meeting developmental goals. Although the importance of inter-rater reliability in conferring rigour in qualitative research is contested (Armstrong et al. 1997; Barbour 2001), it should be acknowledged that lack of use here may have impacted analysis through increasing the interpretive nature of coding.

Despite these limitations, this study raises important issues regarding the perceived positive role of alcohol in peer group formation for this cohort and the implications of this for the university in attempting to reduce alcohol-related harm. Further consideration of approaches to moderating student alcohol use during this period should be explored.

## References

[CIT0001] Ahern, J., S. Galea, A. Hubbard, L. Midanik, and S. L. Syme. 2008. “Culture of Drinking” and Individual Problems with Alcohol Use.” *American Journal of Epidemiology* 167 (9): 1041–1049. doi:10.1093/aje/kwn022. 18310621 PMC9985457

[CIT0002] Armstrong, D., A. Gosling, J. Weinman, and T. Marteau. 1997. “The Place of Inter-Rater Reliability in Qualitative Research: An Empirical Study.” *Sociology* 31 (3): 597–606. doi:10.1177/0038038597031003015.

[CIT0003] Banister, E. N., and M. G. Piacentini. 2006. “‘Binge Drinking-Do They Mean Us?’: Living Life to the Full in Students’ Own Words.” *Advances in Consumer Research* 33: 390–398.

[CIT0004] Barbour, R. S. 2001. “Checklists for Improving Rigour in Qualitative Research: A Case of the Tail Wagging the Dog?” *British Medical Journal* 322 (7294): 1115–1117. 11337448 10.1136/bmj.322.7294.1115 PMC1120242

[CIT0005] Baron, R. S., and N. L. Kerr. 1992. *Group Process, Group Decision, Group Action*. Maidenhead: Open University Press.

[CIT0006] Bassey, M. 1999. *Case Study Research in Educational Settings*. Buckingham: Open University Press.

[CIT0007] Bhattacharya, A. 2016. *Youthful Abandon: Why are Young People Drinking Less?* Institute of Alcohol Studies. Available at: http://www.ias.org.uk/uploads/pdf/IAS%20reports/rp22072016.pdf

[CIT0008] Biederman, J., S. V. Faraone, M. C. Monuteaux, and J. A. Feighner. 2000. “Patterns of Alcohol and Drug Use in Adolescents Can Be Predicted by Parental Substance Use Disorders.” *Pediatrics* 106 (4): 792–797. 11015524 10.1542/peds.106.4.792

[CIT0009] Braun, V., and V. Clarke. 2006. “Using Thematic Analysis in Psychology.” *Qualitative Research in Psychology* 3 (2): 77–101. doi:10.1191/1478088706qp063oa.

[CIT0010] Brooks, O. 2011. “‘Guys! Stop Doing It!’: Young Women’s Adoption and Rejection of Safety Advice When Socializing in Bars, Pubs and Clubs.” *The British Journal of Criminology* 51 (4): 635–651. doi:10.1093/bjc/azr011.

[CIT0011] Brown, R., and S. Murphy. 2018. “Contrasting Staff and Student Views on Alcohol Education Provision in a UK University.” *Drugs: Education, Prevention and Policy (Online)*. doi:10.1080/09687637.2018.1475548. PMC6474724 31058270

[CIT0012] Bryman, A. 2008. *Social Research Methods*. 3rd ed. Oxford: Oxford University Press.

[CIT0013] Buote, V. M., S. M. Pancer, M. W. Pratt, G. Adams, S. Birnie-Lefcovitch, J. Polivy, and M. Gallander Wintre. 2007. “The Importance of Friends: Friendship and Adjustment among 1st Year University Students.” *Journal of Adolescent Research* 22 (6): 665–689. doi:10.1177/0743558407306344.

[CIT0014] Chatterton, P. 1999. “University Students and City-Centres—The Formation of Exclusive Geographies: The Case of Bristol, UK.” *Geoforum* 30: 117–133. doi:10.1016/S0016-7185(98)00028-1.

[CIT0015] Cheng, T. C., and C. C. Lo. 2015. “Change in Adolescents’ Alcohol-Use Patterns, from Non-Drinking to Non-Heavy Drinking or Heavy Drinking.” *Journal of Drug Issues* 45 (4): 447–459. doi:10.1177/0022042615604013.

[CIT0016] Clapp, J. D., A. M. Shillington, and L. B. Segars. 2000. “Deconstructing Contexts of Binge Drinking among College Students.” *American Journal of Drug and Alcohol Abuse* 26 (1): 139–154. 10.1081/ada-100100596 10718169

[CIT0017] Clapp, J. D., M. B. Reed, and D. E. Ruderman. 2014. “The Relationship between Drinking Games and Intentions to Continue Drinking, Intentions to Drive after Drinking, and Adverse Consequences: Results of a Field Study.” *The American Journal of Drug and Alcohol Abuse* 40 (5): 374–379. doi:10.3109/00952990.2014.933838. 25192205

[CIT0018] Collins, S. E., M. Kirouac, E. Taylor, P. J. Spelman, V. Grazioli, G. Hoffman, L. Haelsig et al. 2014. “Advantages and Disadvantages of College Drinking in Students’ Own Words: Content Analysis of the Decisional Balance Worksheet.” *Psychology of Addictive Behaviors* 28 (3): 727–733. DOI:10.1037/a0036354. 25134033

[CIT0019] Conroy, D., and R. de Visser. 2012. “‘Man Up!’: Discursive Constructions of Non-Drinkers among UK Undergraduates.” *Journal of Health Psychology* 18 (11): 1432–1444. doi:10.1177/1359105312463586. 23188922

[CIT0020] Craigs, C. L., B. M. Bewick, J. Gill, F. O’May, and D. Radley. 2011. “UK Student Alcohol Consumption: A Cluster Analysis of Drinking Behaviour Typologies.” *Health Education Journal* 71 (4): 516–526. doi:10.1177/0017896911406967.

[CIT0021] Croom, K., D. Lewis, T. Marchell, M. L. Lesser, V. F. Reyna, L. Kubicki-Bedford, M. Feffer, and L. Staiano-Coico. 2010. “Impact of an Online Alcohol Education Course on Behavior and Harm for Incoming First-Year College Students: Short-Term Evaluation of a Randomized Trial.” *Journal of American College Health* 57 (4): 445–454. doi:10.3200/JACH.57.4.445-454. 19114384

[CIT0022] de Visser, R. O., Z. Wheeler, C. Abraham, and J. A. Smith. 2013. “‘Drinking Is Our Modern Way of Bonding’: Young People’s Beliefs about Interventions to Encourage Moderate Drinking.” *Psychology and Health* 28 (12): 1460–1480. doi:10.1080/08870446.2013.828293. 23947783

[CIT0023] Deaux, K. 2001. “Social Identity.” In *Encyclopaedia of Women and Gender: Sex Similarities and Differences and the Impact of Society on Gender*,edited byN. Levy, Vol. 2, 1059–1068. London: Academic press.

[CIT0024] Dyson, R., and K. Renk. 2006. “Freshman Adaptation to University Life: Depressive Symptoms, Stress and Coping.” *Journal of Clinical Psychology* 62 (10): 1231–1244. doi:10.1002/jclp.20295. 16810671

[CIT0025] Ellison, N. B., C. Steinfield, and C. Lampe. 2007. “The Benefits of Facebook “Friends”: Social Capital and College Students’ Use of Online Social Network Sites.” *Journal of Computer Mediated-Communication* 12 (4): 1143–1168. doi:10.1111/jcmc.2007.12.issue-4.

[CIT0026] Faulkner, S., L. B. Hendry, L. Roderique, and R. Thomson. 2006. “A Preliminary Study of the Attitudes, Triggers and Consequences of Hazardous Drinking in University Students.” *Health Education Journal* 65 (2): 159–169. doi:10.1177/001789690606500206.

[CIT0027] Fisher, S., and B. Hood. 1987. “The Stress of the Transition to University: A Longitudinal Study of Psychological Disturbance, Absent-Mindedness and Vulnerability to Homesickness.” *British Journal of Psychology* 78 (4): 425–441. 3427309 10.1111/j.2044-8295.1987.tb02260.x

[CIT0028] Flyvbjerg, B. 2006. “Five Misunderstandings about Case-Study Research.” *Qualitative Inquiry* 12 (2): 219–245. doi:10.1177/1077800405284363.

[CIT0029] Fox, K., and P. Marsh. 1998. *Social and Cultural Aspects of Drinking: A Report to the European Commission*. Oxford: Social Issues Research Centre.

[CIT0030] Fry, M. L., J. Drennan, J. Previte, A. White, and D. Tjondronegoro. 2014. “The Role of Desire in Understanding Intentions to Drink Responsibly: An Application of the Model of Goal-Directed Behaviour.” *Journal of Marketing Management* 30 (5–6): 551–570. doi:10.1080/0267257X.2013.875931.

[CIT0031] Fuller, A., K. M. Fleming, L. Szatkowski, and M. Bains. 2017. “Nature of Events and Alcohol-Related Content in Marketing Materials at a University Freshers’ Fair: A Summative Content Analysis.” *Journal of Public Health*. doi:10.1093/pubmed/fdx181. 29253185

[CIT0032] Griffin, C., A. Bengry-Howell, C. Hackley, W. Mistral, and I. T. Szmigin. 2009. “The Allure of Belonging: Young People’s Drinking Practices and Collective Identification.” In *Identity in the 21st Century: New Trends in New Times*, edited by M. Wetherell, 213–230. London: Palgrave Press.

[CIT0033] Hebden, R., A. C. Lyons, I. Goodwin, and T. McCreanor. 2015. ““When You Add Alcohol, It Gets that Much Better” University Students, Alcohol Consumption, and Online Drinking Cultures.” *Journal of Drug Issues* 45 (2): 214–226. doi:10.1177/0022042615575375.

[CIT0034] Hepworth, J., C. McVittie, T. Schofield, J. Lindsay, R. Leontini, and J. Germov. 2016. “‘Just Choose the Easy Option’: Students Talk about Alcohol Use and Social Influence.” *Journal of Youth Studies* 19 (2): 251–268. doi:10.1080/13676261.2015.1059928.

[CIT0035] Herring, R., M. Bayley, and R. Hurcombe. 2014. ““But No One Told Me It’s Okay to Not Drink”: A Qualitative Study of Young People Who Drink Little or No Alcohol.” *Journal of Substance Use* 19 (1–2): 95–102. doi:10.3109/14659891.2012.740138.

[CIT0036] Hornsey, M. J., T. Grice, J. Jetten, N. Paulsen, and V. Callan. 2007. “Group-Directed Criticisms and Recommendations for Change: Why Newcomers Arouse More Resistance than Old-Timers.” *Personality and Social Psychology Bulletin* 33 (7): 1036–1048. doi:10.1177/0146167207301029. 17557893

[CIT0037] Institute of Alcohol Studies. (2013). Institute of Alcohol Studies Alcohol and Young People Factsheet. Available at: http://www.ias.org.uk/uploads/pdf/Factsheets/Young%20people%20and%20alcohol%20FS%20May%202013.pdf

[CIT0038] Jayne, M., G. Valentine, and S. L. Holloway. 2011. *Alcohol, Drinking and Drunkenness: (Dis)Orderly Spaces*. Farnham: Ashgate Publishing Limited.

[CIT0039] Johnson, T. J., J. Wendel, and S. Hamilton. 1998. “Social Anxiety, Alcohol Expectancies, and Drinking-Game Participation.” *Addictive Behaviors* 23 (1): 65–79. 9468744 10.1016/s0306-4603(97)00033-6

[CIT0040] Kairouz, S., L. Gliksman, A. Demers, and E. M. Adlaf. 2002. “For All These Reasons I Do.Drink”: A Multilevel Analysis of Contextual Reasons for Drinking among Canadian Undergraduates.” *Journal of Studies on Alcohol* 63 (5): 600–608. 12380857 10.15288/jsa.2002.63.600

[CIT0041] King, N., and C. Horrocks. 2010. *Interviews in Qualitative Research*. London: Sage Publications.

[CIT0042] Larimer, M. E., and J. M. Cronce. 2007. “Identification, Prevention, and Treatment Revisited: Individual-Focused College Drinking Prevention Strategies 1999–2006.” *Addictive Behaviors* 32 (11): 2439–2468. doi:10.1016/j.addbeh.2007.05.006. 17604915

[CIT0043] Lee, R., and S. B. Robbins. 1995. “Measuring Belongingness: The Social Connectedness and Social Assurance Scales.” *Journal of Counseling Psychology* 42 (2): 232–241. doi:10.1037/0022-0167.42.2.232.

[CIT0044] Lee, R. M., K. A. Keough, and J. D. Sexton. 2002. “Social Connectedness, Social Appraisal, and Stress in College Women and Men.” *Journal of Counseling & Development* 80 (3): 355–361. doi:10.1002/j.1556-6678.2002.tb00200.x.

[CIT0045] Lee, R. M., M. Draper, and S. Lee. 2001. “Social Connectedness, Dysfunctional Interpersonal Behaviors, and Psychological Distress: Testing a Mediator Model.” *Journal of Counselling Psychology* 48 (3): 310–318. doi:10.1037/0022-0167.48.3.310.

[CIT0046] LivingStone, A. G., H. Young, A. S. R. Manstead, J. R. Smith, W. R. Louis, and P. W. Schultz. 2011. ““We Drink, Therefore We Are” the Role of Group Identification and Norms in Sustaining and Challenging Heavy Drinking “Culture”.” *Group Processes & Intergroup Relations* 14 (5): 637–649. doi:10.1177/1368430210392399.

[CIT0047] Mallinckrodt, B. 1998. “Student Retention, Social Support, and Dropout Intention: Comparison of Black and White Students.” *Journal of College Student Development* 29 (1): 60–64.

[CIT0048] Mashek, D., J. Stuewig, E. Furukawa, and J. Tangney. 2006. “Psychological and Behavioral Implications of Connectedness to Communities with Opposing Values and Beliefs.” *Journal of Social and Clinical Psychology* 25 (4): 404–428. doi:10.1521/jscp.2006.25.4.404. 21532983 PMC3084011

[CIT0049] McMillan, W. 2013. “Transition to University: The Role Played by Emotion.” *European Journal of Dental Education* 17 (3): 169–176. doi:10.1111/eje.12026. 23815694

[CIT0050] Measham, F., and K. Brain. 2005. “‘Binge’ Drinking, British Alcohol Policy and the New Culture of Intoxication.” *Crime, Media, Culture* 1 (3): 262–283. doi:10.1177/1741659005057641.

[CIT0051] Miles, S., D. Cliff, and V. Burr. 1998. “‘Fitting in and Sticking Out’: Consumption, Consumer Meanings and the Construction of Young People’s Identities.” *Journal of Youth Studies* 1 (1): 81–96. doi:10.1080/13676261.1998.10592996.

[CIT0052] Miller, T., and L. Bell. 2002. “Consenting to What? Issues of Access, Gate-Keeping and ‘Informed’ Consent.” In *Ethics in Qualitative Research*, edited byM. Mauthner, M. Birch, and J. Jessop, 61–75. Abridged. London: Sage Publications.

[CIT0053] Morse, J. M. 1997. ““Perfectly Healthy, but Dead”.: The Myth of Inter-Rater Reliability.” *Qualitative Health Research* 7: 445–447. doi:10.1177/104973239700700401.

[CIT0054] Morton, F., and B. Tighe. 2011. “Prevalence Of, and Factors Influencing, Binge Drinking in Young Adult University Under-Graduates.” *Journal of Human Nutrition and Dietetics* 24: 296–297. doi:10.1111/j.1365-277X.2011.01175_25.x/pdf.

[CIT0055] Newbury-Birch, D., E. Gilvarry, P. McArdle, V. Ramesh, S. Stewart, J. Walker, L. Avery et al. (2009). Impact of Alcohol Consumption on Young People: A Review of Reviews. Department for Children, Schools and Families, HM Government [Online]. Available at: http://dera.ioe.ac.uk/10929/

[CIT0056] Newton, N. 2010. The Use of Semi-Structured Interviews in Qualitative Research: Strengths and Weaknesses. Academia [Online]. Available at: http://www.academia.edu/1561689/The_use_of_semi-structured_interviews_in_qualitative_research_strengths_and_weaknesses

[CIT0057] Nicpon, M. F., L. Huser, E. Hull Blanks, S. Sollenberger, C. Befort, and S. E. R. Robinson Kurpius. 2006. “The Relationship of Loneliness and Social Support with College Freshmen’s Academic Performance and Persistence.” *Journal of College Student Retention: Research, Theory and Practice* 8 (3): 345–358. doi:10.2190/A465-356M-7652-783R.

[CIT0058] Orme, J., and N. Coghill. 2014. “Wasted Potential: The Role of Higher Education Institutions in Supporting Safe, Sensible and Social Drinking among Students.” *Health Education Journal* 73 (2): 192–200. doi:10.1177/0017896912471041.

[CIT0059] Palmer, M. J., P. O’Kane, and M. Owens. 2009. “Betwixt Spaces: Student Accounts of Turning Point Experiences in the First‐Year Transition.” *Studies in Higher Education* 34 (1): 37–54. doi:10.1080/03075070802601929.

[CIT0060] Paschall, M. J., T. Antin, C. L. Ringwalt, and R. F. Saltz. 2011. “Effects of AlcoholEdu for College on Alcohol-Related Problems among Freshmen: A Randomized Multicampus Trial.” *Journal of Studies on Alcohol and Drugs* 72 (4): 642–650. 21683046 10.15288/jsad.2011.72.642 PMC3125887

[CIT0061] Paul, E. L., and S. Brier. 2001. “Friendsickness in the Transition to College: Precollege Predictors and College Adjustment Correlates.” *Journal of Counseling and Development* 79 (1): 77–89. doi:10.1002/j.1556-6676.2001.tb01946.x.

[CIT0062] Polizzotto, M. N., M. M. Saw, I. Tjhung, E. H. Chua, and T. R. Stockwell. 2007. “Fluid Skills: Drinking Games and Alcohol Consumption among Australian University Students.” *Drug and Alcohol Review* 26 (5): 469–475. doi:10.1080/09595230701494374. 17701509

[CIT0063] Raffo, C., and M. Reeves. 2000. “Youth Transitions and Social Exclusion: Developments in Social Capital Theory.” *Journal of Youth Studies* 3 (2): 147–166. doi:10.1080/713684372.

[CIT0064] Rapley, T. 2008. Doing Conversation, Discourse and Document Analysis. London: Sage Publications

[CIT0065] Ray, A. E., J. L. Stapleton, R. Turrisi, and E. Y. Mun. 2014. “Drinking Game Play among First-Year College Student Drinkers: An Event-Specific Analysis of the Risk for Alcohol Use and Problems.” *The American Journal of Drug and Alcohol Abuse* 40 (5): 353–358. doi:10.3109/00952990.2014.930151. 25192202 PMC4263026

[CIT0066] Read, J. P., M. D. Wood, C. W. Kahler, J. E. Maddock, and T. P. Palfai. 2003. “Examining the Role of Drinking Motives in College Student Alcohol Use and Problems.” *Psychology of Addictive Behaviors* 17 (1): 13–23. 12665077 10.1037/0893-164x.17.1.13

[CIT0067] Rice, K. 1992. “Separation-Individuation and Adjustment to College: A Longitudinal Study.” *Journal of Counselling Psychology* 39 (2): 203–213. doi:10.1037/0022-0167.39.2.203.

[CIT0068] Roche, A. M., and K. Watt. 1999. “Drinking and University Students: From Celebration to Inebriation.” *Drug and Alcohol Review* 18 (4): 389–399. doi:10.1080/09595239996257.

[CIT0069] Rosenquist, J. N., J. Murabito, J. H. Fowler, and N. A. Christakis. 2010. “The Spread of Alcohol Consumption Behavior in a Large Social Network.” *Annals of Internal Medicine* 152 (7): 426–433. doi:10.7326/0003-4819-152-7-201004060-00007. 20368648 PMC3343772

[CIT0070] Seaman, P., and T. Ikegwuonu 2010. Drinking to Belong: Understanding Young Adults’ Alcohol Use within Social Networks. Joseph Rowntree Foundation. Available at: http://www.jrf.org.uk/sites/files/jrf/alcohol-young-adults-full.pdf.

[CIT0071] Seaman, P., and T. Ikegwuonu. 2011. “‘I Don’t Think Old People Should Go to Clubs’: How Universal Is the Alcohol Transition Amongst Young Adults in the United Kingdom.” *Journal of Youth Studies* 14 (7): 745–759. doi:10.1080/13676261.2011.588946.

[CIT0072] Snow, P. C., S. Wallace, P. K. Staiger, and B. J. Stolz-Grobusch. 2003. ““As Long as It Doesn’t Spill over into Class”: Harms Arising from Students’ Alcohol Use, and the Role of Policy in Reducing Them.” *International Journal of Drug Policy* 14 (1): 5–16. doi:10.1016/SO955-3959(02)00198.

[CIT0073] Stake, R. E. 1995. *The Art of Case Study Research*. London: Sage Publications.

[CIT0074] Stake, R. E. 2000. “Case Studies.” In *Handbook of Qualitative Research*, edited byN. K. Denzin and Y. S. Lincoln, 435–454. 2nd ed. London: Sage Publications.

[CIT0075] Thombs, D. L., R. Scott Olds, S. J. Bondy, J. Winchell, D. Baliunas, and J. Rehm. 2009. “Undergraduate Drinking and Academic Performance: A Prospective Investigation with Objective Measures.” *Journal of Studies on Alcohol and Drugs* 70 (5): 776–785. 19737503 10.15288/jsad.2009.70.776 PMC2741556

[CIT0076] Tinto, V. 1975. “Dropout from Higher Education: A Theoretical Synthesis of Recent Research.” *Review of Educational Research* 45: 89–125. doi:10.3102/00346543045001089.

[CIT0077] Ward, B. W., and J. Gryczynski. 2009. “Social Learning Theory and the Effects of Living Arrangements on Heavy Alcohol Use: Results from a National Study of College Students.” *Journal of Studies on Alcohol and Drugs* 70 (3): 364–372. 19371487 10.15288/jsad.2009.70.364

[CIT0078] White, A., and R. Hingson. 2013. “The Burden of Alcohol Use: Excessive Alcohol Consumption and Related Consequences among College Students.” *Alcohol Research: Current Reviews* 35 (2): 201–218. 24881329 10.35946/arcr.v35.2.11 PMC3908712

[CIT0079] Workman, T. 2001. “Finding the Meanings of College Drinking: An Analysis of Fraternity Drinking Stories.” *Health Communication* 13 (4): 427–447. 11771805 10.1207/s15327027hc1304_05

[CIT0080] Zamboanga, B. L., J. V. Olthuis, S. R. Kenney, C. J. Correia, K. Van Tyne, L. S. Ham, and B. Borsari. 2014. “Not Just Fun and Games: A Review of College Drinking Games Research from 2004 to 2013.” *Psychology of Addictive Behaviors* 28 (3): 682–695. doi:10.1037/a0036639. 25222171 PMC4356507

